# Involving Health Care Professionals in the Development of Electronic Health Records: Scoping Review

**DOI:** 10.2196/45598

**Published:** 2023-07-10

**Authors:** Theresa Sophie Busse, Chantal Jux, Johannes Laser, Peter Rasche, Horst Christian Vollmar, Jan P Ehlers, Sven Kernebeck

**Affiliations:** 1 Institute of General Practice and Family Medicine (AM RUB) Medical Faculty Ruhr University Bochum Bochum Germany; 2 School of Nursing Saint Elisabeth Group GmbH Catholic Hospitals Rhine-Ruhr Herne Germany; 3 Chair of Didactics and Educational Research in Healthcare Faculty of Health Witten/Herdecke University Witten Germany; 4 Faculty of Health Care Niederrhein University of Applied Sciences Krefeld Germany

**Keywords:** user-centered design, electronic health records, electronic medical records, digital technology, technology development, stakeholder participation

## Abstract

**Background:**

Electronic health records (EHRs) are a promising approach to document and map (complex) health information gathered in health care worldwide. However, possible unintended consequences during use, which can occur owing to low usability or the lack of adaption to existing workflows (eg, high cognitive load), may pose a challenge. To prevent this, the involvement of users in the development of EHRs is crucial and growing. Overall, involvement is designed to be very multifaceted, for example, in terms of the timing, frequency, or even methods used to capture user preferences.

**Objective:**

Setting, users and their needs, and the context and practice of health care must be considered in the design and subsequent implementation of EHRs. Many different approaches to user involvement exist, each requiring a variety of methodological choices. The aim of the study was to provide an overview of the existing forms of user involvement and the circumstances they need and to provide support for the planning of new involvement processes.

**Methods:**

We conducted a scoping review to provide a database for future projects on which design of inclusion is worthwhile and to show the diversity of reporting. Using a very broad search string, we searched the PubMed, CINAHL, and Scopus databases. In addition, we searched Google Scholar. Hits were screened according to scoping review methodology and then examined, focusing on methods and materials, participants, frequency and design of the development, and competencies of the researchers involved.

**Results:**

In total, 70 articles were included in the final analysis. There was a wide range of methods of involvement. Physicians and nurses were the most frequently included groups and, in most cases, were involved only once in the process. The approach of involvement (eg, co-design) was not specified in most of the studies (44/70, 63%). Further qualitative deficiencies in the reporting were evident in the presentation of the competences of members of the research and development teams. Think-aloud sessions, interviews, and prototypes were frequently used.

**Conclusions:**

This review provides insights into the diversity of health care professionals’ involvement in the development of EHRs. It provides an overview of the different approaches in various fields of health care. However, it also shows the necessity of considering quality standards in the development of EHRs together with future users and the need for reporting this in future studies.

## Introduction

### Background

The use of electronic health records (EHRs) is increasing worldwide [1,2]. It has been associated with improvements in health care quality and patient safety [3]. In international literature, different terms are used interchangeably to refer to electronic clinical documentation, such as electronic medical records, electronic patient records, or EHRs [4]. In this paper, we use the term EHRs to refer to different types of electronic documentation of patient health data. EHRs are digitized medical records used in clinical health care within an organization [5]. EHRs are linked to organizations (eg, an EHR that is used by the staff in an intensive care unit [ICU] of a hospital), as opposed to personal health records. Personal health records are characterized by the fact that patients can manage them themselves and provide access to others [6]. EHRs can electronically gather and record both administrative and health-related information as well as store, transmit, and display information from various sources [7]. Traditionally, health-related information in EHRs includes a medical history and medication orders, vital signs, or laboratory results [3,8]. Administrative information may include age, sex, or International Classification of Diseases codes [9]. Depending on the context, EHRs include different submodules, such as medication display, the display of vital signs, or diagnostic information [10]. For example, different content is more critical for work in an ICU than for work in a palliative care unit. Depending on the context, there are EHRs specific to each area of medical care to ensure optimal documentational support [11]. It is useful to ensure that information can be transferred within the units of a hospital and between health care institutions. However, this diversity of records still poses challenges for interoperability [12].

In recent years, technological progress has led to extreme improvements in the field of EHRs in terms of design and functions [13]. EHRs can be used to minimize costs and workload with the help of shared, location independent, and clear documentation [14] and to improve collaboration and coordination between different professions and individuals [15]. In addition to the digitization of previously paper-based documentation, electronic decision support systems and the use of predefined clinical guidelines and standards can support quality improvement based on the latest health care knowledge [3,16].

However, the solitary implementation and use of EHRs in isolation will not guarantee that the quality of care improves. The literature suggests that EHRs with poor usability or functionality may have unintended consequences for their users and patients [17]. For example, the lack of adaption to workflows [18,19] and user needs [20], poor usability [21], and unstructured data sets in EHRs lead to high cognitive demands on users [22]. These aspects are associated with work-related stress, fatigue, and burnout for the main user groups of EHRs: nurses [23,24] and physicians [25-27]. Furthermore, poor usability has been associated with patient harm [28]. For example, it can make it difficult for health care providers to access necessary medical data for the treatment of patients or lead to misinterpretation of available data. This can lead to misdiagnosis, incorrect treatment, or unsuitable medication for a patient’s condition. This can put the patient’s health and well-being at risk [28]. Therefore, user acceptance is essential for successful implementation, actual use, and user satisfaction of EHRs [29]. Expected usefulness, technical concerns, technical problems, and expected workflow challenges can facilitate or hinder technology acceptance [30].

To promote international joint development projects, globally valid standards have been drawn up. For example, there is International Organization for Standardization (ISO) 9241-210, which focuses on the ergonomics of human-system interaction. It is an important standard for classifying and demanding usability engineering measures in product development processes such as the development of EHRs. Unfortunately, these standards are often only partially complied with, leading to the various abovementioned problems.

In addition, the involvement of users is necessary to adapt EHRs to the needs of health care professionals and to ensure their acceptance [31]. This is increasingly being addressed, resulting in an expansion of projects involving future users in EHR development. Existing reviews have focused on the involvement of users in technology development. In recent years, several reviews have been published to address the involvement of users in the development of different health-related technologies [32,33]. The focus of these reviews has been, on the one hand, on the involvement of different user groups, such as older people [34-39], people living with dementia [40], or patients with chronic diseases [41]. Specific to these groups is the fact that their cognitive and physiological characteristics must be addressed in the development of digital technologies. On the other hand, reviews cover different use cases of technologies such as mobile health [42], serious digital games for health promotion [43], or for the treatment of depression [41]. In addition, other reviews cover more general aspects of user involvement for the development of health-related technologies [32,33]. Despite the empirical evidence supporting the need for user involvement in the development and implementation of EHRs, this topic is largely excluded from the reviews. For example, in 3 reviews covering generic aspects of user involvement, no study focused on EHRs [32,33,44]. Different approaches such as participatory design or co-design are common practices to involve users in the development of new technologies. For example, on the one hand, participatory design actively and creatively involves both users and designers and thus includes different individual qualifications [31,45]. This approach can be defined as “...a process of investigating, understanding, reflecting upon, establishing, developing, and supporting mutual learning between multiple participants in collective ‘reflection-in-action’. The participants typically undertake the two principle roles of users and designers where the designers strive to learn the realities of the users’ situation while the users strive to articulate their desired aims and learn appropriate technological means to obtain them” [45]. On the other hand, an approach such as co-design is defined as an “active collaboration between stakeholders in the design of solutions to a pre-specified problem” [46]. Although these 2 approaches are often used interchangeably and synonymously, they differ in how much choice is given to users in the development of a technology. It can be assumed that the participatory design approach gives users more influence than the co-design approach.

### Aim

In existing studies, the design of the methodology varies depending on resources, time period, and technology. When using participatory design or co-design methods, methodological choices must be made [46]. For the planning of similar research projects and a sensible use of diverse methods, it is crucial to provide an overview. Within the framework of a scoping review, we therefore investigated which forms of user involvement have been used to date, under what circumstances, and with what results. The result can also be used to facilitate guidelines for the involvement of health professionals in the development of EHRs. The overview in the metadata table in Multimedia Appendix 1 [47-115] is intended to be particularly helpful in this regard. In addition to the range of possibilities, specific classifications can also be made as to which method is helpful and for which objective.

The review was guided by the question: “How are health care professionals involved in the development of EHRs?”

## Methods

### Overview

The *Methods* section is reported as recommended by the PRISMA (Preferred Reporting Items for Systematic Reviews and Meta-Analyses) 2020 statement [33]. The presentation of this scoping review is based on the methodological specifications by Peters et al [116].

This methodology was developed using an a priori scoping review protocol [117]. The decision to use a scoping review methodology was based on the identification of a gap in current knowledge [117] and the need for an overview of different methods without any assessment. The result should be a narrative account with a focus on the different ways and methods of involving end users in the development of EHRs.

The methodology of scoping reviews is gaining popularity, particularly in the field of health care [118]. Whereas systematic reviews aim to synthesize collate empirical evidence on a focused research question and present the evidence from the reviewed studies [119], scoping reviews map the existing literature on a topic area [120]. In addition, scoping reviews provide a descriptive overview [121] and are therefore an appropriate method for addressing the research question.

This review aimed to provide an overview of the existing ways and methods of user involvement in the development of EHRs in the literature. The four specific objectives of this review were (1) to conduct a systematic search of the published literature for studies focusing on user involvement in the development of EHRs, (2) to present the characteristics and range of methods used in the identified manuscripts, (3) to explore the reported challenges and limitations of the methods, and (4) to make recommendations for the further development of the approach to the development of EHRs and to improve the consistency with which these types of studies are conducted and reported.

Planning for the review began in January 2021. The review was conducted and evaluated from June 2021 to April 2022. Four people were involved in carrying out the review (JL, CJ, SK, and TSB). JL and CJ had experience in conducting (scoping) reviews. CJ, SK, and TSB worked with prospective users to develop an outpatient EHR, an inpatient EHR, and a cross-sectoral EHR for pediatric palliative care with future users.

### Eligibility Criteria

Studies were eligible for inclusion if they described the involvement of health care professionals in the development of EHRs. This explicitly included studies that examined a specific EHR. However, excluded studies focused on the general workload resulting from the use of different EHRs in different institutions or other parameters related to different EHRs, regardless of their design. Articles published in languages other than English were excluded. Manuscripts that described a process without performing it were excluded from the scoping review. Gray literature was not included because of the focus on research projects, although this was included in the scoping review methodology [122].

SK and JL formulated the inclusion and exclusion criteria and discussed them with TSB and CJ. The inclusion and exclusion criteria are listed in Textboxes 1 and 2.

**Inclusion criteria.**

**Languages**

- English

**Publication period**

- 2011-2021

**Format**

- Full text available

**Study design**

- Empirical studies on the development of an electronic health record (EHR) or modules or submodules where health care professionals are involved
- Needs assessment
- Requirements testing or evaluation
- Qualitative, quantitative, and mixed methods studies

**Forms of publication**

- Papers published in a scientific journal

**Product**

- EHR
- Submodules or modules integrated into an EHR
- Same EHR in different stages of development

**Development phases**

- Pending testing or a major effectiveness study
- Implementation
- Evaluation

**Setting**

- All settings in health and social care

**Participants in development process**

- Health care professionals, even if other groups of people are involved

**Exclusion criteria.**

**Languages**

- Languages other than English

**Publication period**

- Before 2011

**Format**

- Abstract only or full text not available

**Study design**

- Reviews
- Randomized controlled trials

**Forms of publication**

- Study protocols
- Conference papers
- Gray literature
- Books
- Bachelor thesis, master thesis, or similar works

**Product**

- Decision support systems
- Personal health records
- Other technologies (integrated apps)
- Comparison of different electronic health records in one survey
- Electronic health record for education or training purposes
- Hardware-specific evaluations

**Development phases**

- No restriction was made with regard to the development phase

**Setting**

- No setting was excluded

**Participants in development process**

- Exclusively patients or other users
- Trainees or students in the health care sector without patient contacts

### Information Sources

The search was carried out in the PubMed, CINAHL, and Scopus databases. A supplementary search was carried out in Google Scholar. The final search was performed by JL and SK on March 17, 2021, using the search strings from Multimedia Appendix 2. The forward-and-backward citation tracking [123] was then carried out by JL using Scopus and Google Scholar.

### Search Strategy

First, an initial limited search was conducted in a selection of relevant databases to analyze possible terms in the title and abstract to identify keywords describing the articles. This was followed by a search of all databases using all identified keywords. SK and JL formulated the basic idea of the review and conducted the initial searches. Afterward, SK, JL, and TSB developed the search terms. The search terms used were based on two main categories: (1) search terms around the term EHRs and corresponding synonyms as well as Medical Subject Headings terms (PubMed) and subject headings (CINAHL) were used, and (2) search terms around the term participatory design with corresponding synonyms and Medical Subject Headings terms or subject headings were used. The search strings for PubMed, CINAHL, Google Scholar, and Scopus are shown in Multimedia Appendix 2.

The search in Google Scholar used a substantially shortened search string, as the search engine cannot process longer, complex search strings. This resulted in several results that did not meet the inclusion criteria. Therefore, using Google Scholar’s *sort by relevance* function, only the first 250 results were checked for eligibility, of which 47 were selected.

### Selection Process

All citations were imported into the bibliographic manager EndNote (Clarivate), and duplicate citations were automatically removed, with further duplicates removed if found later in the process. The citations were then imported into the software [124] to subsequently check the relevance of the titles and summaries and to characterize the data of the full articles. Rayyan provided blinded checking and automatically displayed matching inclusions, exclusions, and conflicts after blinding was turned off.

First, the titles and abstracts were checked by SK and JL to ensure compliance with the inclusion criteria. Differences were discussed with TSB. Subsequently, TSB and JL screened titles and abstracts for the forward-and-backward citation tracking results, and the differences were discussed with CJ.

All citations deemed relevant after title and abstract screening were obtained for subsequent review of the full-text article. For articles that could not be obtained through institutional holdings that were available to the authors, attempts were made to contact the authors of the source and request the article. In addition, articles were requested via interlibrary loan.

SK and JL screened the full texts; the differences were discussed with TSB. TSB and JL screened the full texts of the forward-and-backward citation tracking results; the differences were discussed with CJ.

At this stage, studies were excluded if they did not meet the eligibility criteria. After reviewing approximately 25 articles independently, the reviewers met to resolve any conflicts and to ensure consistency among the reviewers and with the research question and purpose [125]. The excluded studies were appropriately labeled with the reason for exclusion to improve traceability.

### Data Analysis

Categories were formed deductively. This was based on a systematic review by Vandekerckhove et al [33] that focused on electronic health interventions. The aim of the review by Vandekerckhove et al [33] was to report and justify participatory design methods in empirical eHealth studies for further development of the methodology. The decision to follow this review was based on its comprehensive presentation and its fit for the research question pursued here. However, the categories were supplemented by inductive categories that emerged from reviewing the material. The categories can be named as “factual categories” according to Kuckartz [126], designating specific facts in the included studies. All codes were reviewed, coded, and discussed in regular meetings by TSB, CJ, and SK.

### Categories for Syntheses

Owing to the diversity of study designs and the research questions, a quality assessment was not performed. Following the approach of Vandekerckhove et al [33], an assessment of the sufficiency and design of reporting was conducted. To improve comprehensibility, the inductive categories were supplemented by key questions (based on the definitions of the categories that were created and constantly refined during the analysis process) and served to represent the collected data items. This was partly based on the categories in the study by Vandekerckhove et al [33], whose review dealt with eHealth interventions. For example, category 1 in this review was developed based on the category “eHealth intervention” by Vandekerckhove et al [33] and category 3—study participants—was based on the category “stakeholder types” by Vandekerckhove et al [33]. Category 4—methods and materials—was based on the category “tools of participatory design” by Vandekerckhove et al [33]. The other categories were derived from the material itself, as described earlier in the *Data Analysis* section. This resulted in the following division, which was used to structure the method representation:

1. Focus and scope of the studies: What is mentioned about the characteristics of the EHR to be developed and the stage of the technology (prototype, already implemented EHR)? What was the aim of the studies?
2. Setting: Where did the involvement in the development of the EHR take place?
3. Study participants: Who was involved in the development? Which characteristics were mentioned when describing the study participants?
4. Methods and materials: Which study design was used? Which terminology was used to describe the involvement process? Which methods were used? Are there any physical materials used in the process? How often were participants involved in the process (involvement counts as renewed involvement if it takes place at a later point in time and contributes to the further development of the technology)?
5. Frameworks, theories, and guidelines: What approaches have been used and influence the choice of methods? Which approaches were used only within the data analysis or a specific method without influencing the choice of methods for the entire study? Which design guidelines were mentioned that influenced the basic logic of the EHR design?
6. Competencies of the researchers: What competencies do researchers contribute in terms of development?

## Results

### Study Selection

**Figure 1:**
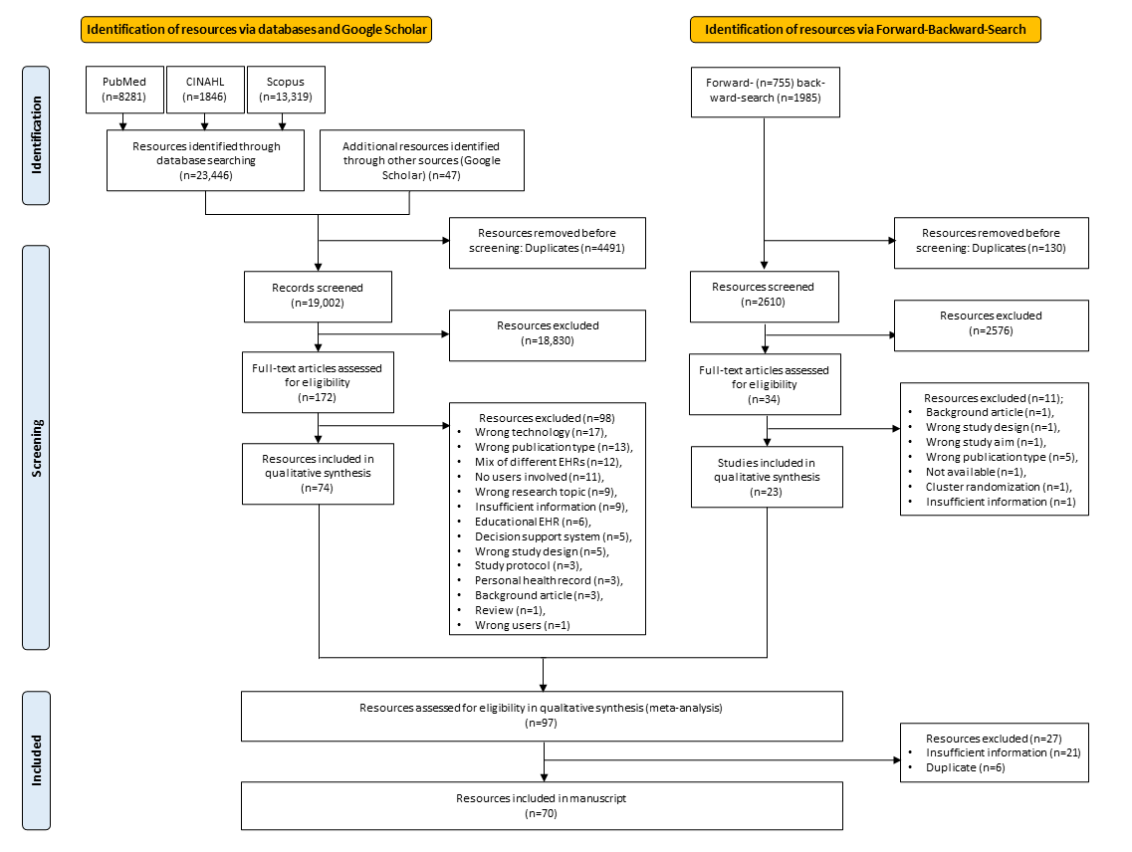
Flow diagram of the study selection process. EHR: electronic health record.

The study selection is described in Figure 1.

The initial search resulted in a total of 23,446 hits (PubMed: n=8281, 35.32%; CINAHL: n=1846, 7.87%; Scopus: n=13,319, 56.81%). In addition, 47 records were extracted from other sources (Google Scholar) after screening the first 25 pages of approximately 7710 results. From a total of 23,446 hits from the initial search and these 47 additional records, 19,002 (81.04%) hits remained after duplicate reduction.

Of these 19,002 articles, 18,830 (99.09%) articles were excluded during title and abstract screening. The remaining 172 texts were subjected to full-text screening, resulting in a total of 74 titles.

The forward-and-backward citation search yielded a total of 2769 hits (755 by forward citation and 1985 by backward citation). Automatic deduplication reduced the number of hits to 2665. Manual duplicate reduction led to a final result of 2625 hits. After title and abstract screening (34 texts remaining), full-text screening was carried out, resulting in 23 articles.

These 23 articles from the forward-and-backward citation search were included in the final assessment along with the previous 74 studies from the initial search. Of these 97 studies, 27 (28%) studies were excluded because of insufficient information and duplicates. The remaining 70 articles were included in the evaluation and can be found in the metadata table in Multimedia Appendix 1.

### Study Characteristics

Multimedia Appendix 1 includes the metadata of the included studies.

### Results of Syntheses

#### Focus and Scope of the Studies

The included studies targeted different objectives in EHR development, which can be divided into 8 groups. Some of the studies focused primarily on eliciting users’ needs and wants toward EHRs. This included (1) studies to collect general information on user needs and preferences [47-50,63,84,88,93,104-106,112] or (2) studies focusing on factors for implementation [60,69,85,111]. Other studies were oriented toward actual implementation and (3) described pilot-testing [90] or (4) the overall design process [65,76,77,114]. Further studies were based on refining the existing content such as (5) studies that focused on the redesign of EHRs or prototypes [57,62,66] or (6) studies that included system improvement and further development [57,91,95,99]. In addition, studies have examined implemented EHRs, which were (7) studies in terms of overall satisfaction or acceptance [51,64,67,68,79-81,86,96,107] or (8) studies focusing on terms of usability or system performance [52-54,58,59,61,​^70^-^75^,^78^,^87^,^89^,^92^,^94^,^97^,^98^,^100^-^103^].

The included studies covered different forms of EHRs and development, which were divided into 4 different categories:

1. Information needs for subsequent programing of EHRs were the area of research of 4 studies [47-50]. In these studies, the authors addressed the information needs in EHRs from the perspective of clinicians. For example, Acharya et al [47,51] gathered information needs for oral health information for an EHR, and Ellsworth et al [49] surveyed the information needs for a neonatal intensive care EHR.
2. In total, 5 studies reported on prototypes or mock-ups [52-56]. For example, Belden et al [57] addressed a clinical note prototype, and Horsky et al [54] developed a prototype of an EHR that allowed clinicians to complete a summary for outpatient visits.
3. Entire EHRs, including all submodules were addressed in 30 studies [56,58-86]. For example, Nolan et al [73] examined information use and workflow patterns for an EHR in an ICU.
4. Individual modules of an EHR were addressed in 31 studies [51,57,70,87-113]. These studies focused on individual modules of an EHR rather than an entire EHR. In this category, for example, Aakre et al [87] focused on a module for the automatic calculation of a sequential organ failure assessment calculator in sepsis detection, and Ahluwalia et al [88] focused on dyspnea assessment for palliative care.

#### Setting

The included studies were conducted in the context of different health care settings. A total of 25 studies were conducted in an unspecified hospital setting [60,62,65,67,70-72,75-78,80-85,​^89^,^91^,^92^,^99^,^100^,^111^,^114^]. Furthermore, 8 studies were conducted in the context of ICUs [49,55,56,58,​^63^,^68^,^73^,^87^,^93^,^115^], 6 were conducted in the context of family medicine [52,59,101,102,109,110], 6 were conducted in primary care hospitals [53,61,97,98,104,105], 5 were conducted in outpatient or clinic settings [54,57,74,95,108], and 3 were conducted in tertiary hospitals [50,86,107]. Two studies each were conducted in a dental clinic [47,51], in a palliative care setting [88,96], in emergency departments [90,103], in the gynecological and antenatal settings [64,66], and in the context of mental health or psychiatry settings [48,69]. One study each was conducted in the setting of home care [94], older adult care [79], community health [106], cancer centers [112], and childcare [113].

#### Study Participants

The participants in the included studies comprised a total of 15 different professions (Table 1). Physicians were involved in 76% (53/70) of the studies, whereas nurses were involved in 40% (28/70) of the studies. Pharmacists were involved in 10% (7/70) of the studies, physiotherapists were involved in 6% (4/70) of the studies, social workers were involved in 4% (3/70) of the studies, and medical assistants were involved in 3% (2/70) of the studies. In 16% (11/70) of the studies, the user groups were not specified. Demographic characteristics such as age, sex, and education were described in 21 studies [53,57,67-70,73,75,79,81,84,91-93,96,98,103,105,106,111,113,114]. Moreover, in 3 studies, the authors provided a brief description of demographic characteristics [64,74,97]. For example, one study provided a description of the demographic characteristics as follows: “The sample consisted of 21 female participants and 9 male participants, with a proportion of 70% female and 30% male” [64]. The remaining studies did not describe the demographic characteristics of the participants.

In 10% (7/70) of the included studies, participants received financial compensation for taking part in the study. The following amounts were paid to the participants: US $100 gift card—25 to 45 minutes [92], US $100 gift card [68], US $50 per hour [75], US $100 per 2 hours [76], €40 (US $43) per hour [96], and US $100 per hour [56]. In one study, a US $25 gift card for a restaurant was offered [59].

In addition to the characteristics of the participants, the number of participants in each study was divided into 7 categories (Table 2).

**Table 1:** User groups included in the studies (n=70).

User group	Studies that included this user group, n (%)	Studies
Physicians	52 (74)	[48-50,52-60,62-71,73,75,76,78,79,82,83,86,88,89,91-93,95-97,100-105,107-110,112-115]
Nurses	29 (41)	[48-50,60,64,65,69,72,74,78-81,84,85,88,93,94,96,99,100,102,103,105-107,111,113]
Pharmacists	7 (10)	[65,78,79,82,85,90,91]
Physiotherapists	4 (6)	[60,78,79,97]
Social workers	3 (4)	[79,85,93]
Medical assistants	2 (3)	[74,106]
Psychologists	1 (1)	[85]
Physician assistants	1 (1)	[106]
Managers	1 (1)	[85]
Medical office assistants	1 (1)	[98]
Midwives	1 (1)	[66]
Community health agents (CHA)	1 (1)	[48]
Primary care providers	1 (1)	[92]
Medical secretaries	1 (1)	[60]
IT departments, hospital’s IT	1 (1)	[60]
Not specified	11 (16)	[47,51,57,61,62,77,78,85,87,105,113]

**Table 2:** Number of participants per study (n=70).

Range for the number of participants	Studies, n (%)	Studies
1-10	15 (21)	[54-56,63,77,82,83,89,95,97,98,100,108,109,115]
11-20	19 (27)	[52,53,58-61,66,74-76,87,88,92,96,99,103,104,110]
21-30	12 (17)	[49,57,64,67-69,72,73,90,102,107,114]
31-40	5 (7)	[71,79,84,94,105]
41-50	5 (7)	[48,65,80,91,101]
51-100	5 (7)	[51,78,106,111,113]
>100	9 (13)	[47,50,62,70,81,85,86,93,112]

#### Methods and Materials

First, the *basic methodology* of the studies was examined. Overall, 36% (25/70) of the studies used mixed methods design [54,55,57,60,61,66,72,75,78,79,81,83,84,90,92,94,95,98,100,​^102^,^103^,^105^,^108^,^109^,^111^,^113^]. Moreover, 31% (22/70) of the studies used a qualitative design [49,50,52,56,59,62,64,​^69^-^71^,^73^,^74^,^77^,^86^,^87^,^91^,^96^,^99^,^104^,^107^,^114^,^115^], whereas 31% (22/70) of the studies used a quantitative design [47,48,​^51^,^53^,^58^,^63^,^65^,^67^,^68^,^76^,^80^,^82^,^85^,^88^,^89^,^93^,^97^,^99^,^101^,^106^,^110^,^112^].

The wide variance of *terminology* in relation to the involvement of users in technology development already mentioned at the beginning is also reflected in the articles included. The terminology here describes the literal naming of the method by the authors of the studies themselves, regardless of how it was conducted. The largest proportion of studies (44/70, 63%) did not include a designation of methodology [47,51,52,55-62​,^64^,^66^,^67^,^69^,^71^,^72^,^76^-^86^,^88^,^90^-^93^,^95^,^98^-^101^,^104^,^106^,^107^,^110^,​^111^,^115^]. For example, in 23% (16/70) of the studies, the authors of the respective manuscripts described the methodological approach as “user-centered design” [54,57,68,70,73-75,79,87,93,97,102,103,108,109,114]. In one of the studies, it was only given as a keyword and not in the manuscript [97]. In terms of frequency, the following terms were used: “participatory design” in 6% (4/70) of the studies [48,63,65,96], “co-design” in 3% (2/70) of the studies [94,105], and “iterative rapid design involving providers” [53], “end-user design” [49], “multidisciplinary design” [61] and “human-centered design” [113] in 1 study each.

The *frequency* with which users were involved in the development was examined. Involvement counts as renewed involvement if it takes place at a later point and contributes to the further development of the technology (for example, several surveys for the iterative refinement of a prototype). In 57% (40/70) of the studies, users were included once [47,49-51,55,56,58,59,69-73,75-77,80,82-84,88,91-93,95-97,​^99^-^101^,^103^-^106^,^108^-^111^,^114^,^115^]. An involvement of users at 2 points was investigated in 24% (17/70) of the studies. Moreover, 9% (6/70) of the studies reported 3 times of user involvement, 4% (3/70) of the studies reported 4 times of involvement, 23% (2/70) of the studies reported 6 times of involvement, 1% (1/69) study reported 5 times of involvement, and 1% (1/69) study reported 9 times of involvement.

In some of the studies, a *foundation* of the study was provided before the actual (further) development of the EHR. This included, for example, literature reviews [47,61,63,68,90], pilot-testing of the design [52], pilot-testing of the survey [81] or interview guide [47,51,68], a review of 12 different EHRs [57] as well as training with the software in advance [76,77,83,103,107], and the presentation of learning videos [91].

A common *method of data collection and involvement of health care professionals* was to test a prototype as a walkthrough using think-aloud technique [52,55,56,58,59,71,74-77,83,89,​^90^,^92^,^93^,^95^-^100^,^103^-^105^,^108^-^110^,^113^-^115^]. As part of the walkthrough methodology, various programs (eg, Morae) have been used to record audio or screen displays, mouse clicks, and keyboard [52,54,74-76,83,92,94,97,100,103,109,113,114]. Eye-tracking software (eg, Tobii T120 eye tracker) was used [52,59,71,75]. A related method, the near-live testing, was used in one study [89].

Another common method used were the questionnaires. In addition to individually created surveys [47,49,50,60,64,66,70,78,79,83,86,101-103,106,107,113], various existing questionnaires were used (Table 3).

Some of the studies used web-based questionnaire tools (eg, Survey Monkey) [47,50,61,69,70,86,113].

Individual semistructured interviews [48,53-55,60,63,65,67,​^68^,^70^,^72^,^79^,^80^,^82^-^85^,^87^,^88^,^90^,^93^,^94^,^96^-^98^,^102^,^104^-^106^,^110^,^111^,^113^,^114^] and group interviews and focus group discussions [48,60,62,63,67] were conducted. In some of the studies, design workshops were held with various users [53,57,65,90,110].

One method that was often combined with interviews was observation. This involved observing health care professionals as they used an EHR to conduct documentation [60,63,66,73,79,85,87,90,94,102,113,114]. This includes observations in both a clinical and a study setting.

The use of mock-ups was another common method in the studies. This contained paper prototypes [57,90,94] and web-based prototypes using different prototyping tools (eg, HipMunk) [57,87,90,93,94,105,113,114].

**Table 3:** Questionnaires used in the studies.

Questionnaire	Focus of the questionnaire	Studies using the questionnaire
Baylor EHR^a^ user experience survey	Measuring user experience following EHR implementation	[69]
Canada Health Infoway System and Use Assessment Survey	Measuring user adoption and use as well as information and system quality	[81]
Computer Systems Usability Questionnaire	Measuring satisfaction of users with computer system usability	[87]
Information System Use Instrument	Measuring nurses’ information systems use	[81]
Keystroke-level model GOMS	Predict or estimate the time for completing a task in software	[59]
Nasa Task Load Index	Measuring perceived workload	[52,58,59,92,113]
Physician Documentation Quality Instrument-9	Assessing the quality of physician electronic documentation	[75]
Post-Study System Usability Questionnaire	Measuring the perceived satisfaction	[100]
Questionnaire for user interaction satisfaction (short form)	Measuring the subjective satisfaction with the human-computer interface	[94]
Single Ease Question	Assessing the difficulty of a task	[74,90]
System Usability Scale	Measuring the usability	[52,55,70,72,75,77,78,94,100,103,108]
Technology Acceptance Model Questionnaire	Measuring likelihood of technology acceptance	[61]
Usability Assessment	Measuring usability	[72]
Workflow Integration Survey	Measuring workflow integration	[81]

^a^EHR: electronic health record.

Less frequently used methods include document analysis [48,85] and extraction of routine data from the EHR for analysis [60,61,70,74,111,113].

An overview of the respective methods by study aim is available in the metadata table in Multimedia Appendix 1.

#### Frameworks, Theories, and Guidelines

In the following studies, *frameworks* are understood as approaches that frame the entire research project and influence the choice of methods and their structure. In these studies, the updated DeLone and McLean framework [127] for evaluating information systems success was used once [60]. The design science framework [128] was used once to develop prototype dashboards [94]. In the same study, the tasks, users, representations, and functions framework [129] was used to structure the usability evaluation [94]. Falah et al [64] used the plan-do-study-act cycle [130] to facilitate the implementation process. In addition, Dziadzko et al [86] used the define-measure-analyze-improve-control quality measurement [131] for implementation measurement. The social science approach of lightweight ethnography [132] was used to design the study by Chruscicki et al [90]. Owing to limited time, the predesigned sample, and particular research questions, one study [67] used framework analysis [133] as a framework. Sockolow et al [79] used the health information technology research-based evaluation framework [134] as a framework to design their study and as a theory to merge qualitative and quantitative data. In addition, in 7% (5/70) of the studies, the authors referred to ISO 9241-210 in their theoretical background [59,70,75,113,114]. However, ISO 9241-210 was not used as a theoretical framework in any of the incident studies.

*Theories* are understood to be those approaches that were used exclusively within the data evaluation or specific analytic method but did not influence the choice of methods for the entire study as a whole. Kernebeck et al [96] used the Unified Theory of Acceptance and Use of Technology [135] to evaluate think-aloud sessions. Wawrzyniak et al [82] used the critical incident technique by Flanagan [136] to design interview protocols. The interview guide in another study [105] was based on the diffusion of innovations theory [137] and complementary ones. The human factors model Systems Engineering Initiative for Patient Safety 2.0 [138] was used by Cohen et al [106] for data collection. The Cognitive Load Theory [139] was described as important and used for evaluation by Curran et al [92]. In addition, the attention capacity model [140], which focuses on mental effort, was used as a theoretical background by Mosaly et al [71]. The technology acceptance model [141] was used to design a questionnaire [113].

In addition, design *guidelines* were mentioned that influenced the basic logic of the EHRs design. This includes the ergonomics of activity [142] mentioned in one study [48], the suggested time and motion procedures [143] in another study [73], and usability heuristics by Nielsen [144] in 1 study [74]. Another framework used was the spiral model for software development [145] in addition to the EHR system user interface framework of the Veterans Affairs Computerized Patient Record System [146] in 1 study [95]. The data-knowledge-information-wisdom framework [147] was named as an important component of informatics in nursing by Nation et al [72].

#### Competencies of Researchers

In addition, all studies were screened for the description of the competencies of the researchers who conducted the studies. A distinction can be made between competences related to software knowledge (eg, usability experience and software programming) or competences related to knowledge of the context of use (eg, previous experience of working with EHRs in clinical settings) or methodological skills in the area of data collection (qualitative interviews, surveys). In 30% (21/70) of the studies, the authors briefly described the competencies of the researchers [49,52,53,60,63,68,70,74,75,82,88,93,97,99,​^103^,^104^,^106^,^109^,^110^,^112^,^113^]. Examples of these descriptions were that the researchers described themselves as “experienced in qualitative research” [106] and “the research team included three academic researchers and two clinical nurses” [99].

## Discussion

This review aimed to provide an overview of the existing methods of user involvement in the literature for developing and evaluating EHRs.

### Principal Findings

The review had four objectives: (1) to conduct a systematic search of the published literature for studies focusing on user involvement in the development of EHRs, (2) to present the characteristics and range of methods used in the identified manuscripts, (3) to explore the reported challenges and limitations of the methods, and (4) to make recommendations for further developing the approach and improving the consistency with which they are conducted and reported. Therefore, the main focus of the review was to examine in which settings which participants were involved with which methods and materials and which frameworks were used. Furthermore, the frequency and design of the development and an overview of the competences of the respective researchers involved in the development were examined. To the best of our knowledge, this is the first review to describe the methodological aspects for involving health care professionals in the development of EHRs.

The characteristics of EHRs addressed in the included studies covered a variety of different aspects. On the one hand, a large number of studies addressed a comprehensive EHR, whereas on the other hand, many studies addressed only individual modules of an EHR. The wide range of characteristics in this review was largely because of the broad inclusion criteria, which were designed to provide a comprehensive picture of the methodological aspects of professional involvement in the development of EHRs. This leads to a better description of the complex field of EHRs and their methodological aspects. However, this sometimes makes it difficult to compare the interventions. In terms of setting, it was found that most of the included studies were conducted in general hospitals and ICUs. The authors suggest analyzing studies in a specific setting and how the participants are involved there in the future to cover the methodological aspects, such as in ICU or in palliative care. Future studies could also focus on the individual modules of an EHR, for example, medication modules.

In terms of participants, the studies mainly included physicians and nurses. In terms of multidisciplinary care, it would be desirable for all health care professionals (including physiotherapists, occupational therapists, speech therapists, social workers, nursing assistants, etc) to record their activities and observations in joint documentation and to be able to view them mutually. In addition, sharing EHRs can facilitate communication [148] (eg, by sending messages within a program and assigning tasks). With this in mind, it is surprising that only these 2 professional groups were so intensively involved. It would be desirable for further studies to include all professional groups and to design EHRs to meet their needs. However, most of the studies did not specify which health care professionals were involved in the development. This was partly because of an imprecise naming of the participants and partly because of the lack of naming. Future studies should specifically describe the demographic characteristics of the participants, which may lead to a better assessment of the results [33,36]. This is important because demographic variables have a strong influence on the acceptance of EHRs and the level of competence in using EHRs and digital health technologies in general [149,150]. Therefore, it is recommended that study investigators collect key demographic variables from participants and present them in tabular form to improve the interpretation of the results.

The methodology of the studies was balanced between qualitative, quantitative, and mixed methods. However, in 63% (44/70) of the studies, no terminology was used to describe the design of user involvement in more detail. This adds to the imprecision of the presentation in terms of the level of involvement and a qualitative assessment of the methodology. Although 23% (16/70) of the included studies that mentioned user-centered design as an approach will be examined to see if this was really implemented, most of the studies remain vague about user involvement. This again supports the broad search strategy of the review but also points to qualitative ambiguities of implementation.

The frequency of user involvement varied widely, and in most of the studies (40/70, 57%), users were involved only at one point. This shows that a true participatory design or co-design, as it is called for, is rather rare and fuels the suspicion of sham participation, where user requirements are collected but no iteration is performed to test the fit. Another problem with user involvement in development is that it often occurs at only one point in the development of new technologies. This problem is often referred to as “project-based temporality” in the involvement of users [151]. Therefore, it is recommended that users are involved in the development of new technologies at all stages of development over a longer period [151].

Most of the methods vary widely. Think-aloud approaches were often used to obtain user feedback on an EHR. The continuation of this method, near-live testing, which also increases the likelihood of a good fit, was used in only one study. In the case of questionnaires, individual, nonvalidated questionnaires were frequently used, which reduced the quality of the results. The most commonly used questionnaires were mainly oriented toward usability (System Usability Scale) in 16% (11/70) of the studies and the cognitive load (Nasa Task Load Index) in 7% (5/70) of the studies. Cognitive load refers to the amount of mental effort required by a person to perform a specific task and the associated required capacity of the human working memory [152]. It is imperative to consider cognitive load in the development of EHRs and in the implementation of appropriate solutions in clinical practice, as cognitive load has been linked to the development of burnout and distress [27]. It is recommended that user involvement studies use a mix of methods from the fields of telling, making, and enacting [153]. This emphasizes the impact of user involvement through the exchange of current and future practices and the sharing of needs (telling). For successful user involvement, it is crucial that future users develop something (making), contributing to the existence and design of a new technology. In addition, by involving users, it should be possible to transfer ideas into reality by creating a simulation to test them [153]. Accordingly, different questions require different methods from each field, but a mixed methods approach ensures a diversity of perspectives.

Frameworks, theories, and guidelines were very rarely used. Moreover, the results showed a rather low level of consideration of the theoretical underpinnings to the detriment of the quality of the studies. This is particularly problematic because the use of such frameworks can structure the development and make the replicability of results between different studies comparable. Furthermore, it is problematic that a large number of studies did not refer to theories and models. This would also provide a theoretical basis for the development and make the quality of the results more comprehensible [32]. It is particularly surprising that only 9% (6/70) of the studies referenced to ISO standards. These standardizes the process for the development of new software. It would be useful to refer to these standards in future studies and to highlight the stage of development of the respective technology [154,155].

The researchers’ skills have rarely been documented. A multidisciplinary research and development team should consist of individuals with different skills from different health care disciplines, methodological disciplines, and social disciplines. This allows for optimal design and support of different stakeholders during development and implementation [156]. Similar to the sometimes imprecise description of demographic characteristics of the study participants, the skills of the investigators should be described. Owing to the interdisciplinary nature of technology development research projects, it would be advisable to have multidisciplinary teams and to identify the respective competencies and experience in technology development.

### Limitations

To be able to interpret these results, it is necessary to describe several limitations of this study. First, the search strategy was limited by its focus on empirical, scientifically published work. The involvement of health care professionals in the development of EHRs may not always be published in scientific journals. Therefore, this review is a first step on the topic of involving health care professionals in the development of EHRs. In further studies, it might be interesting to include gray literature and databases with a focus on technology-oriented research and engineering (eg, Institute of Electrical and Electronics Engineers). However, the heterogeneity of the quality of the publications must be taken into account. In addition, EHRs are mostly developed by large digital technology companies. It can be assumed that these companies often involve users in the development but do not produce publications or perform actual research. Therefore, it can be assumed that there was publication bias. It would be necessary in the future to survey such large companies on how they involve users in the development of EHRs.

Second, it should be noted that the screening process was limited by the definitions of user involvement, which accordingly shaped both the search terms and the inclusion and exclusion criteria. Although a wide range of terms were used, it cannot be ruled out that individual manuscripts that were coherent in terms of content, and therefore would have led to different results, were not included because of the lack of used terminology.

Third, it should be taken into account that some studies published their results in several manuscripts and did not briefly review the entire development or implementation process. In our evaluation, we were only able to consider the described frequency of user involvement from the information provided in the included manuscripts. However, it is possible that the included manuscripts each report only a subset of the study project; whereas in the overall study project, users were much more frequently involved.

Finally, one of the findings, namely the sometimes-low transparency of reporting, also directly points to a limitation in terms of analysis and conclusions—drawing conclusions on the basis of reporting in manuscripts has limited validity, as there is no way to ensure that the actual methodological considerations and intentions correspond to what was presented in the manuscript.

### Further Research

Further research can help to improve the methodological framework for involving health care professionals in the development of EHRs. Particular attention should be paid to the rationale for the methodological choices. It is also crucial to combine different methods from the fields of telling, making, and enacting; involve users at several points in time to avoid sham participation; and strive for maximum user orientation. The growing interest in “design through design research” [157] should be encouraged but with conditions to promote high-quality developments. Little knowledge can be gained from publications with low reporting quality in terms of transferability and quality assessments. It would be useful for studies to report the aspects more precisely. Specific reporting guidelines for reporting the results of technology development studies would be helpful, as is the case for many other types of studies [158]. Process evaluations should be used in a standardized manner to improve study quality.

In addition, in future studies, it would be necessary to examine in more detail the outcomes of the participatory design with users. In this context, questions should be answered regarding the specific outcomes that have been improved by the involvement of users. How these outcomes were measured and how, for example, improvements to the software were evaluated in different iterations should also be analyzed.

### Conclusions

Studies involving health care professionals in the development of EHRs have used various approaches. This paper provides an overview of the approaches in different fields of development with the inclusion of diverse users. Often, however, there is no specific approach, framework, or theory underlying the procedure and there is missing or inaccurate information in the reporting.

## Appendix

Multimedia Appendix 1 Metadata of the included studies.

Multimedia Appendix 2 Search string.

## Abbreviations

EHR: electronic health record
ICU: intensive care unit
ISO: International Organization for Standardization
PRISMA: Preferred Reporting Items for Systematic Reviews and Meta-Analyses
