# Supplementary material for: Involving Health Care Professionals in the Development of Electronic Health Records: Scoping Review
Source: JMIR Hum Factors. 2023 Jul 10;10:e45598. doi: 10.2196/45598 (PMC10366971; doi:10.2196/45598)
Supplement: Multimedia Appendix 1 [file humanfactors_v10i1e45598_app1.docx]

Involving Healthcare Professionals in the Development and Evaluation of Electronic Health Records – A Scoping Review

# Appendix 2: Metadata of the included studies

| **Publi-cation year** | **Country** | **Authors** | **Title** | **Technology** | **Methods** | **User/sample/further information** |
| --- | --- | --- | --- | --- | --- | --- |
| 2017 | USA | Aakre CA; Kitson JE; Li M; Herasevich V | Iterative User Interface Design for Automated Sequential Organ Failure Assessment Score Calculator in Sepsis Detection | Modul of an EMR (sequential organ failure assessment score calculator) | Observation  Interviews  UX mockup review  Evaluation questionnaire | 14 internal medicine residents (interface development)  12 clinicians (evaluation): 1 resident, 4 fellows, 7 attending physicians |
| 2011 | USA | Acharya A; Mahnke A; Chyou PH; Rottscheit C; Starren JB | Medical providers’ dental information needs: a baseline survey | Medical-dental integrated EHR | Survey | 417 medical care providers  Grouped based on role and specialty  Roles:  1. Physician, Surgeon, Anesthesiologist, Medical Director, Department Chair, Resident and Nurse Practitioner  2. Certified Nurse, Nurse Midwives, Licensed Practical Nurses and Registered Nurse  3. Managers and Others  Specialties:  1. Surgery; 2. Cardiology; 3. Emergency Medicine; 4. Primary Care; 5. Oncology; 6. Pediatrics; 7. Neurology; 8. Women’s Health/Obstetrics-Gynecology; 9. Other Specialties. |
| 2017 | USA | Acharya A; Shimpi N; Mahnke A; Mathias R; Ye Z | Medical care providers’ perspectives on dental information needs in electronic health records | Medical-dental integrated EHR | Focus groups  Thematic analysis methodology | 65 participants  Roles: medical assistant, manager, physician assistant, registered nurse, physician, nurse practitioner, licensed nurse practitioner, appointment coordinator, divisional operation technician, medical lab technician, ancillary services coordinator, certified medical assistant, certified nurse midwife, secretary, transcription |
| 2013 | USA | Ahluwalia SC; Leos RL; Goebel JR; Asch SM; Lorenz KA | Provider approaches to palliative dyspnea assessment: implications for informatics-based clinical tools | EMR-based dyspnea assessment tool | Interviews | 13 palliative care program managers:  10 physicians  3 advanced practice nurses |
| 2011 | USA | Ahmed A; Chandra S; Herasevich V; Gajic O; Pickering BW | The effect of two different electronic health record user interfaces on intensive care provider task load, errors of cognition, and performance | 2 EHR interfaces | Crossover study  Task performance simulation  NASA task load index | 20 providers: 6 attending physicians, 14 residents/fellows |
| 2020 | Lebanon | Al Ghalayini M; Antoun J; Moacdieh NM | Too much or too little? Investigating the usability of high and low data displays of the same electronic medical record | 2 EMR display | Task performance testing  Think-aloud  NASA task load index | 13 residents (6 males, 7 females; age m=29 years (SD=2,68); experience in the family medicine department m=2,46 years (SD=1); self rating of proficiency with the EMR m=4 (SD=0,39) (1 poor - 5 excellent)) |
| 2017 | USA | Belden JL; Koopman RJ; Patil SJ; Lowrance NJ; Petroski GF; Smith JB | Dynamic Electronic Health Record Note Prototype: Seeing More by Showing Less | Note design in an EHR | Comparative usability testing  NASA task load index | 16 physicians (56% female; 81% faculty, 13% of whom internal medicine physicians; 94% with over 5 years of experience using the EHR) |
| 2019 | USA | Belden JL; Wegier P; Patel J; Hutson A; Plaisant C; Moore JL; Lowrance NJ; Boren SA; Koopman, RJ | Designing a medication timeline for patients and physicians | Medication timeline visualization (in an EHR) | research and analysis of  existing medication lists and timelines, design workshops, team meetings to refine prototype, small pilot evaluation of the design | advisory panel of 12 subject matter experts inhuman factors, pharmacology, medicine, and nursing  evaluation group of 23 clinicians (physicians, nurses, pharmacists), human factors researchers,  usability practitioners, and volunteers from the EHR vendor com-  munity: (52% male; 48% female; age 26% 24-34 years, 17% 35-44 years, 22% 45-54 years, 30% 55-64 years, 4% 65-74 years; computer skill 4% beginner, 78% intermediate, 17% expert; experience as healthcare provider 26% 0-5 years, 13% 6-10 years, 13% 11-20 years, 48% 21 years or more) |
| 2013 | Denmark | Bossen C; Jensen LG; Udsen FW | Evaluation of a comprehensive EHR based on the DeLone and McLean model for IS success: approach, results, and success factors | Comprehensive EHR | Ethnographic observation  Questionnaire  Interviews  Focus group interviews | Physicians, nurses, medical secretaries, physiotherapists   - 13 individual interviews: 4 physicians; 3 nurses; 3 physiotherapists; 3 medical secretaries - 7 focus-group interviews with (no Number provided) with Physicians, nurses, medical secretaries, physiotherapists |
| 2014 |  | Briggs B, Carter-Templeton H | Electronic Health Record Customization: A Quality Impromevement Project | EHR | Literature review, routine data review, online questionnaire, real world test | 6 physicians, 5 nurse practitioners, 2 physician assistants, 25 medical assistants |
| 2011 | USA | Brokel JM; Ochylski S; Kramer JM | Re-engineering workflows: Changing the life cycle of an electronic health record system | EHR | Post-implementation group interviews | 16 physicians  >100 end users |
| 2020 | USA | Calzoni L, Clermont G, Cooper G, Visweswaran S, Hochheiser H | Graphical Presentations of Clinical Data in a Learning Electronic Medical Record | EHR in an intensive care unit | Literature review, observation, focus groups | 5 physicians for observations, 5 physicians for focus groups |
| 2011 | USA | Carrington JM; Effken JA | Strengths and limitations of the electronic health record for documenting clinical events | EHR | semi-structured interviews | 37 registered nurses |
| 2014 | [Canada, USA?] | Chrimes D; Kitos NR; Kushniruk A; Mann DM | Usability testing of Avoiding Diabetes Thru Action Plan Targeting (ADAPT) decision support for integrating care-based counseling of pre-diabetes in an electronic health record | Module for EHR software interface (clinical decision support tool) | Usability testing using think-aloud (Phase I)  Near-live clinical simulation (Phase II) | 8 primary care providers (I), thereof 4 residents  5 primary care providers (II) |
| 2016 | Canada | Chruscicki A; Badke K; Peddie D; Small S; Balka E; Hohl CM | Pilot-testing an adverse drug event reporting form prior to its implementation in an electronic health record | Adverse drug events documentation platform (implemented in an EMR) | Lightweight ethnography  Observation, shadowing | 6 clinical pharmacists |
| 2021 | Canada | Clark RE; Milligan J; Ashe MC; Faulkner G; Canfield C; Funnell L; Brien S; Butt DA; Mehan U; Samson K; Papaioannou A; Giangregorio L | A patient-oriented approach to the development of a primary care physical activity screen for embedding into electronic medical records | physical activity screen for EMR | semi-structured interviews, draft of physical activity screening questions for the prototype, iterative discussions, usability testing using paper version with short debrief interview | interviews:  9 physicians/nurse practitioners  7 nurses  11 other healthcare professionals  3 other stakeholders  4 patients  usability testing:  2 physicians |
| 2014 | USA | Clarke MA, Steege LM, Moore JL, Koopman RJ, Belden JL, Kim MS | Determining Primary Care Physician Information Needs to Inform Ambulatory Visit Note Display | Ambulatory Visit Note Display | Interviews, walkthrough | 13 family medicine physicians, 2 internal medicine physicians |
| 2020 | USA | Cohen DJ; Wyte-Lake T; Dorr DA; Gold R; Holden RJ; Koopman RJ; Colasurdo J; Warren N | Unmet information needs of clinical teams delivering care to complex patients and design strategies to address those needs | EHR | interviews and observations of care teams in community health centers | 65 clinicians  168 of support staff  of 9 community health centers |
| 2019 | [USA?] | Cooler J; Ross CA; Robert S; Linder L; Ruhe AM; Philip A | Evaluation and optimization of take-home naloxone in an academic medical center | Pharmacist screening tool within the EHR | Performance evaluation  Discussion | Stakeholders:  Clinical pharmacists  Pharmacy department stakeholders  Key physician stakeholders  44 patients (representing a typical clinical pharmacist patient load):  Male, n (%): 21 (47.7)  Mean age, y (interquartile range): 62.1 (23.5)  Admitting diagnosis, n (%): Other 15 (34.1); Infection 11 (25); Respiratory 11 (25); Cardiovascular 7 (15.9)  Median length of stay, d: (range) 6 (1-77) |
| 2012 | Scotland | Cresswell KM; Worth A; Sheikh A | Integration of a nationally procured electronic health record system into user work practices | EHR | semi-structured interviews, non-participant observation, field notes, hospital documents | 66 interview-participants from junior and senior nurses and doctors, allied health professionals and administrative staff  interviews with 14 stakeholders outside the hospital |
| 2020 | USA | Curran RL; Kukhareva PV; Taft T; Weir CR; Reese TJ; Nanjo C; Rodriguez-Loya S; Martin DK; Warner PB; Shields DE; Flynn MC; Boltax JP; Kawamoto K | Integrated displays to improve chronic disease management in ambulatory care: A SMART on FHIR application informed by mixed-methods user testing | chronic disease management application in EHR | retrospective think-aloud sessions (capturing timing, keyboard and mouse use), parts of NASA-TLX survey, perceived workload measure | 10 physicians, 1 nurse practitioner, 2 physician assistants |
| 2021 | USA | Desai AV, Agarwal R, Epstein AS, Kuperman GJ, Michael CL, Mittelstaedt H, Connor M, Bernal C, Lynch KA, Ostroff JS, Katz B, Corrigan KL, Kramer D, Davis ME, Nelson JE | Needs and Perspectives of Cancer Center Stakeholders for Access to Patient Values in the Electronic Health Record | EHR | Semistructured interviews | 110 stakeholders from medical oncology (n=8), surgical oncology (n=5), radiation oncology (n=2), hospital medicine (n=9), critical care medicine (n=3), interventional proceduralists (n=3), supportive care (n=5), psychiatry (n=3), subspeciality medicine (n=3), medical trainees (n=12) |
| 2021 |  | Desai AV, Michael CL, Kuperman GJ, Jordan G, Mittelstaedt H, Epstein AS, Connor M, Villar RPB, Bernal C, Kramer D, Davis ME, Chen Y, Malisse C, Markose G, Nelson JE | A novel patient values tab for the electronic health record: A user-centered approach | EHR (Patient-Values Tab) | Interviews, mock-ups, walkthrough | “over 100 stakeholders” |
| 2020 | Brazil | do Carmo Alonso CM; de Lima AN; Oggioni B de MP, Teixeira MR, Oliveira EP, Couto MCV, Duarte FJCM | Contributions of activity ergonomics to the design of an electronic health record to support collaborative mental care of children and youth: Preliminary results | EHR | Demand analysis (interviews, spreadsheets)  Content analysis principles according to Mayring | Primary care center:  18 Community Health Agents  3 General Practitioners  3 Nurses  Psychosocial care center:  6 Psychologists  2 Psychiatrists  3 Social Workers  2 Nurses  2 Pedagogue  1 Occupational therapist  1 Speech therapist |
| 2019 | USA | Dowding D; Merrill JA; Barrón Y; Onorato N; Jonas K; Russell D | Usability Evaluation of a Dashboard for Home Care Nurses | Dashboard (of an EHR) | Contextual interviews  Usability evaluation  Rapid feedback | 32 nurses in total:  2 nurses as co-designers (contextual interviews and usability evaluation)  10 nurses (rapid feedback)  20 nurses (usability evaluation)  22 nurses who completed the usability evaluation:  20 female (91%)  Mean age: 51 years (SD=10.0)  32% white non-Hispanic; 32% Asian; 23% African-American; 14% Hispanic, Other  78% had 10 or more years of nursing experience |
| 2016 | USA | Dziadzko MA; Herasevich V; Sen A; Pickering BW; Knight AMA; Moreno Franco P | User perception and experience of the introduction of a novel critical care patient viewer in the ICU setting | EMR interface | surveys before and after implementation of novel EMR interface | 246 surveys before and 115 after from physicians, nurse practitioners, physician assistants, respiratory therapists, registered nurses and other critical care medical staff from ICU |
| 2014 | USA | Ellsworth MA; Lang TR; Pickering BW; Herasevich V | Clinical data needs in the neonatal intensive care unit electronic medical record | neonatal intensive care EMR | web-based survey | 8 attending physicians  2 neonatal fellows  4 neonatal nurse practitioners  9 pediatric residents |
| 2020 | Jordan | Falah J; Alfalah SFM; Halawani S; Abulebbeh M; Muhaidat N | EMR for obstetric emergency department and labour ward in Jordan University Hospital | Tablet based EMR system | Interviews  Questionnaire | 4 consultant obstetricians  15 residents  5 interns  6 nurses  21 (70%) female, 9 (30%) male |
| 2012 | USA | Farri O; Rahman A; Monsen KA; Zhang R; Pakhomov SV; Pieczkiewicz DS; Speedie SM; Melton GB | Impact of a prototype visualization tool for new information in EHR clinical documents | Information visualization tool within a prototype EHR system | Clinical scenario and routine task simulation (observations, think-aloud)  Interviews | 8 clinicians (intern level physicians):  4 males, 4 females  aged 21 - 30 years |
| 2013 | Spain | Gascón F; Herrera I; Vázquez C; Jiménez P; Jiménez J; Real C; Pérez F | Electronic health record: Design and implementation of a lab test request module | Lab test request module EHR | two surveys | first survey: 22 laboratories  second survey: 66 clinical users (35 urban area, 31 rural area) |
| 2019 | Portugal | Grenha Teixeira JG; Pinho NF; Patrício L | Bringing service design to the development of health information systems: The case of the Portuguese national electronic health record | National EHRs | Interviews  Focus groups  Workshops  Internal design meetings | 172 stakeholders (doctors, nurses, pharmacists, citizens)  Per service design process stage:  Exploration: 48 total, 13 (interviews), 35 (focus groups)  Ideation and Reflection: 87 total, 20 (internal design meetings), 32 + 35 (participatory workshops)  Implementation and Evaluation: 37 total, 37 (interviews) |
| 2019 | Japan | Helou S; Abou-Khalil V; Yamamoto G; Kondoh E; Tamura H; Hiragi S; Sugiyama O; Okamoto K; Nambu M; Kuroda T | Understanding the Situated Roles of Electronic Medical Record Systems to Enable Redesign: Mixed Methods Study | EMR | Observation  Survey | 3 obstetricians, 6 midwives (observations)  5 obstetricians, 10 midwives (survey):  Experience with the EMR system:  Obstetricians: 1, 1, 5, 7, 7 years  Midwives: 1, 3, 3, 3, 5, 5, 6, 12, 12, 13 years |
| 2014 | USA | Herasevich V; Ellsworth MA; Hebl JR; Brown MJ; Pickering BW | Information needs for the OR and PACU electronic medical record | EMR | survey | 133 attending physicians  259 certified registered nurse anesthetists  54 physicians-in-training consisting of current anesthesia residents and fellows  72 student registered nurse anesthetists |
| 2013 | Mexico | Hernández-Ávila JE; Palacio-Mejía LS; Lara-Esqueda A; Silvestre E; Agudelo-Botero M; Diana ML; Hotchkiss DR; Plaza B; Sanchez Parbul A | Assessing the process of designing and implementing electronic health records in a statewide public health system: the case of Colima, Mexico | EHR (SAECCOL) | Interviews  Focus groups | 27 participants (interviews) N (%):  Type of actor: Federal level officials 4 (14.8); State level officials 7 (25.9); Hospital and health center managers 10 (37.0); IT staff 6 (22.2)  Sex: Male 20 (74.1); Female 7 (25.9)  Age: 20–39 years 14 (51.9); 40 years or more 13 (48.1)  Training: Physician 17 (63.0); IT specialist 6 (22.2); Other 4 (14.8)  35 participants (focus groups):  Type of health center: Pioneer 16 (45.7); Recently implemented 19 (54.3)  Sex: Male 19 (54.3); Female 16 (45.7)  Age groups: 20–39 years 22 (62.9); 40 years or more 13 (37.1)  Time using SAECCOL: None 3 (8.6); Less than 2 years 27 (77.1); 2 years or more 5 (14.3)  Training: Physician 31 (88.6); Nurse 3 (8.6); Other 1 (2.9) |
| 2016 | [USA?] | Horsky J; Ramelson HZ | Development of a cognitive framework of patient record summary review in the formative phase of user-centered design | Prototype of an ambulatory EHR interface | Informal discussions  Open card sorting  Walkthrough think-aloud  Interviews | Physicians (discussions)  9 clinicians:  10+ years of experience as primary care physicians |
| 2016 | USA | Hultman G; Marquard J; Arsoniadis E; Mink P; Rizvi R; Ramer T; Khairat S; Fickau K; Melton GB | Usability Testing of Two Ambulatory EHR Navigators | EHR ambulatory navigator | Task completion  Single ease question | 8 resident physicians in training:  2nd to 4th year  Experience with EHR: average EHR user 6; expert EHR user 2 |
| 2021 | Germany | Kernebeck S; Busse TS; Jux C; Meyer D; Dreier LA; Zenz D; Zernikow B; Ehlers JP | Participatory design of an electronic medical record for paediatric palliative care: A think-aloud study with nurses and physicians | EMR | think aloud sessions + interview | 10 nurses  6 physicians  from pediatric palliative care  (6 of them with experience in professional use of EMR) |
| 2021 | USA | Khairat S; Coleman C; Teal R; Rezk S; Rand V; Bice T; Carson SS | Physician experiences of screen-level features in a prominent electronic health record: Design recommendations from a qualitative study | Key screen features within an EHR | semi-structured interviews based on literature review and expert feedback | 25 ICU physicians:  Average age: 33.2 years (SD=6.1)  Average experience with EHR system: 4.2 years (SD=1.3)  Clinical Role:  11 Residents; age 29.0 (1.4); exp. 4.0 (0.4)  9 Fellows; age 32.7 (0.5); exp. 5.7 (0.9)  5 Attendings; age 44.0 (6.5); exp. 3.8 (0.4)  Gender:  13 Females; age 31.5 (3.1); exp. 4.0 (1.0)  12 Males; age 34.9 (7.6); exp. 4.3 (1.4) |
| 2019 | USA | King AJ; Cooper GF; Clermont G; Hochheiser H; Hauskrecht M; Sittig DF; Visweswaran S | Using machine learning to selectively highlight patient information | Learning EMR | showing a set of patient cases to physicians, who selected relevant data items for morning rounds  statistical models applied to new patient cases and users review highlighted data | reviewers were ICU physicians ( fellows and attending physicians) trained in critical care medicine  training phase: 11 reviewers (spending 3.0-10.0 years since medical school graduation, spending 0.3-7.0 years in ICU, spending 26-42 weeks per year in ICU)  evaluation phase: 12 reviewers (spending 3.0-11.0 years since medical school graduation, spending 0.6-4.0 years in ICU, spending 28-44 weeks per year in ICU)  5 reviewers participated in both phases |
| 2018 | USA | King AJ; Cooper GF; Hochheiser H; Clermont G; Hauskrecht M; Visweswaran S | Using Machine Learning to Predict the Information Seeking Behavior of Clinicians Using an Electronic Medical Record System | Learning EMR | review of patient cases using tasks | 9 ICU fellows  2 faculty attendings  from the Department of Critical Care Medicine  (7 male, 5.5 years average experience since graduating from medical school, 1.8 years average of ICU experience) |
| 2015 | USA | King AJ; Cooper GF; Hochheiser H; Clermont G; Visweswaran S | Development and Preliminary Evaluation of a Prototype of a Learning Electronic Medical Record System | Learning EMR | physicians review patient cases, results are used to train logistic regression models, preliminary usability study | collecting training cases: 1 physician  usability study: 4 fellows from Department of Critical Care Medicine |
| 2011 | USA | Koopman RJ, Kochendorfer KM, Moore JL, Mehr DR, Wakefield DR, Yadamsuren B, Coberly JS, Kruse RL, Wakfield BJ, Belden JL | A diabetes dashboard and physician efficiency and accuracy in accessing data needed for high quality diabetes care |  |  |  |
| 2015 | USA | Koopman RJ; Steege LMB; Moore JL; Clarke MA; Canfield SM; Kim MS; Belden JL | Physician information needs and electronic health records (EHRs): Time to reengineer the clinic note | EHR | cognitive task analysis, review of acute and chronic care visit notes and highlighting them, semi-structured interview afterwards | 16 primary care physicians, practicing in community clinics associated with a medical school department of family and community medicine and a division of general internal medicine  sampling for maximum variation in sex, years in medical practice, experience with EHR |
| 2017 | [USA?] | Lipford K; Jones S; Johnson K | Needs assessment of an electronic health record at an inpatient psychiatric hospital | EHR | User experience survey | 27 participants:  Gender: Male 5 (18.5%); Female 22 (81.5%)  Age in years: 26-29 1 (3.7); 30-35 1 (3.7); 40-45 3 (11.1); 46-49 3 (11.1); 50-55 5 (18.5); 56-59 3 (11.1); 60-65 7 (25.9); 66-70 4 (14,8)  Physician 1 (3.7); Nurse Practitioner 4 (14.8); Registered Nurse 21 (77.8); Licensed Practical Nurse 1 (3.7)  Comfort level with home computer use: slightly comfortable 1 (3.7); Moderately comfortable 8 (29.6); Very comfortable 18 (66.7) |
| 2017 | Argentina | Luna DR; Lede DAR; Rubin L; Otero CM; Ortiz JM; García MG; Rapisarda RP; Risk MR; Quirós FGB | User-centered design improves the usability of drug-drug interaction alerts: A validation study in the real scenario | drug-drug-interaction alerts in EHR | original interface and participatory design version were shown in allocated groups, metadata was analyzed + survey + interviews | Physicians working for more than one year in the hospital  traditional alert: 168 physicians, aged 28-34, 57% female, 2-4 years seniority  participatory design: 142 physicians, aged 29-34, 56 female, 1-4 years seniority  interviews: 5 traditional and 7 participatory design interface users |
| 2017 | Argentina | Luna DR; Rizzato Lede DA; Otero CM; Risk MR; Quirós FGB | User-centered design improves the usability of drug-drug interaction alerts: Experimental comparison of interfaces | Alert system in an EHR | First phase: interviews,  contextual observations,  clinical vignettes, usability testing  Second phase: experimental interface test | 24 physicians (first phase):  Fields: outpatient, critical inpatient, non-critical inpatient  30 physicians (second phase):  Age in years m=34.3 (SD=3.4); Female 56,7% (N=17); Graduated in years 9.6 (2); EHR use in years 6.7 (0.9)  Outpatient (10):  Age in years 34.2 (3.4); Female 60 (6); Graduated in years 9.5 (2.2); EHR use in years 6.8 (1.1)  Critical Inpatient (10):  Age in years 36 (1.2); Female 50 (5); Graduated in years 10.6 (0.8); EHR use in years 6.6 (0.8)  Non-critical Inpatient (10)  Age in years 32.7 (4.1); Female 60 (6); Graduated in years 8.3 (2.3); EHR use in years 6.6 (0.8) |
| 2016 | USA | Mishuris RG; Yoder J; Wilson D; Mann D | Integrating data from an online diabetes prevention program into an electronic health record and clinical workflow, a design phase usability study | EHR | two rounds of semi-structured interviews: (1) perspectives on integrating lifestyle data, rough mockup, (2) fully developed prototype | primary care providers from the section of general internal medicine and department of family medicine  first round: 10 primary care providers  (7 women, 3 men, 8 physicians, 3 nurse practitioners, 14.6 years in practice average, 7 very comfortable with EHR, 2 comfortable, 1 uncomfortable)  second round: 5 primary care providers (that had already participated in round one)  (1 men, 4 women, 4 physicians, 1 nurse practitioner, 14.4 yeats in practice average, 2 very comfortable with EHR, 2 comfortable, 1 uncomfortable) |
| 2019 | USA | Mosaly PR; Guo H; Mazur L | Toward Better Understanding of Task Difficulty during Physicians’ Interaction with Electronic Health Record System (EHRs) | EHR | Task performance  Eye movements and screen videos capturing | 38 resident physicians:  14 Internal Medicine: Post Graduate Year (PGY) 1-4; 9 female, 5 male  4 Family Medicine: PGY 1-4; 2 female, 2 male  9 Pediatrics: PGY 1-3; 7 female, 2 male  5 Surgery: PGY 1-5; 3 female, 2 male  6 Other: PGY 1-5, 3 female, 3 male  Total: PGY 1: 10; 2: 8; 3: 11; 4: 6; 5: 3; 24 female, 14 male |
| 2019 | USA | Nation J; Wangia-Anderson V | Applying the Data-Knowledge-Information-Wisdom framework to a Usability Evaluation of Electronic Health Record System for Nursing Professionals | EHR | System Usability Scale  Usability Assessment Survey  Open-ended questions | 28 nurses:  Degree: bachelor’s 64%; associate 29%; master`s 7%  Employment: full-time 68%; part-time 5; PRN 5  Nursing experience 1-51 years, m=22.32 years |
| 2020 | Canada | Neudorf B; Giangregorio L; Morita P | Insights From an Usability Review of an Electronic Medical Record–Integrated Physical Activity Counseling Tool for Primary Care | EHR-integrated physical activity counseling tool | Think-aloud testing  Near-live scenario  Interviews | 5 primary care providers:  2 physical therapists, 2 family physicians, 1 specialist in an interprofessional primary care clinic  Age m=48 years (38-57)  Years of practice m=22 (5-30)  Self-rated familiarity with EMR 9/10 (8-10)  Self-rated technological proficiency 8/10 (6-9) |
| 2017 | USA | Nolan ME; Siwani R; Helmi H; Pickering BW; Moreno-Franco P; Herasevich V | Health IT usability focus section: Data use and navigation patterns among medical ICU clinicians during electronic chart review | EHR interface | Direct observation  Workflow analysis | 24 ICU clinicians:  10 attending physicians, 7 fellows, 7 advanced practice providers (nurse practitioners and physician assistants)  Years in clinical practice: 1-34; median 7.5; interquartile range 4-13.5  Usual ICU practice (n=19): medical 16 (84%), mixed medical/surgical 3 (16%)  Primary specialty: Pulmonary and critical care 14 (58%); Critical care - internal medicine 1 (4%); Critical care - anesthesiology 2 (8%); APP training (critical care) 7 (29%)  Familiarity with existing EHR software (n =19): Beginner 2 (11%); Intermediate 7 (37%); Advanced 10 (53%)  (demographic data missing for some participants) |
| 2020 | Kenya | Oyugi B; Makunja S; Kabuti W; Nyongesa C; Schömburg M; Kibe V; Chege M; Gathu S; Wanyee S; Sahal M | Improving the management of hypertension and diabetes: An implementation evaluation of an electronic medical record system in Nairobi County, Kenya | EMR | semi-structured interviews, quantitative analysis, trend analysis, critical evaluation, costing | 52 interviewees  facility in-charges:8  clinical officers: 25  nursing officers: 5  record officers/data clerks: 10  laboratory technologists: 1  sub-county medical officer/non-communicable diseases focal persons: 3  46% 24 years old and below  37% 25-35 years  17% above 35  length of computer use  19% 5 years and below  60% 6 years and above  21% not specified |
| 2020 | USA | Pierce R, Eskridge B, Rehard L, Ross B, Day M, Belden J | The Effect of Electronic Health Record Usability Redesign on Annual Screening Rates in an Ambulatory Setting | EHR | Walkthrough, Single Ease Questionnaire, routine data | 12 participants (9 nurses and 1 medical assistant) |
| 2016 | USA | Press A; DeStio C; McCullagh L; Kapoor S; Morley J; Conigliaro J | Usability Testing of a National Substance Use Screening Tool Embedded in Electronic Health Records | Clinical workflow integrated tool within an EHR | Usability testing  System usability scale survey | 6 medical office assistants (MOAs):  Years worked as MOA: m=4.8 (2-14)  Age: m=34.3 years (23-46)  Years of medical experience: m=8.3 (3-16)  Experience with computer decision support tools: 3 yes, 3 no  Years of use of the current EMR: 2-4 |
| 2017 | USA | Rizvi RF; Marquard JL; Seywerd MA; Adam TJ; Elison JT; Hultman GM; Harder KA; Melton GB | Usability Evaluation of an EHR’s Clinical Notes Interface from the Perspective of Attending and Resident Physicians: An Exploratory Study | Clinical note documentation and clinical note viewing interface in an EHR | Scenario-based usability testing | 14 physicians:  6 attendings; 8 residents, excluding interns  Age 29-43; 8 female, 6 male  Clinical experience (years): (≤5) 12; (>10) 2  Technology experience: 1 less; 11 somewhat, 2 very  Years of using Epic: (<5) 9; (5-10) 4; (>10) 1  Epic proficiency: 9 average, 5 proficient |
| 2013 | USA | Rogers ML; Sockolow PS; Bowles KH; Hand KE; George J | Use of a human factors approach to uncover informatics needs of nurses in documentation of care | Nursing information system (module of an EHR) | Scenario-based evaluation technique | 12 registered nurses  “To protect their anonymity no  socio-demographics were collected.” |
| 2017 | USA | Schall MC Jr; Cullen L; Pennathur P; Chen H; Burrell K; Matthews G | Usability Evaluation and Implementation of a Health Information Technology Dashboard of Evidence-Based Quality Indicators | EHR functional dashboard | Focus group (reviewing display style)  Focus group (discussing data elements)  Task performance evaluation | Care provider (focus group 1)  Nurse managers, physicians, hospital quality professionals (focus group 2)  3 pairs of nurses, 1 physician from medical-surgical areas (task performance evaluation) |
| 2014 | USA | Senathirajah Y; Kaufman D; Bakken S | The clinician in the driver’s seat: Part 2 - Intelligent uses of space in a drag/drop user-composable electronic health record | User-composable EHR system (MedWISE) | Observation  Real case simulation  Screen layout preparation | 13 clinicians:  Resident physicians, attending physicians, 1 physician assistant:  Service at NYP-CUMC (hospital) m=2.5 years; experience in their fields m=3.3 years; experience using WebCIS (EHR) m=2.4 years; experience using other EHRs m=2.7 years  Self-rated computer knowledge: 8 above average, 1 expert |
| 2013 | Malawi | Shah KG; Slough TL; Yeh PT; Gombwa S; Kiromera A; Oden ZM; Richards-Kortum RR | Novel open-source electronic medical records system for palliative care in low-resource settings | Open-source EMR system | Training  Usability tests (task completion)  System usability scale | 17 staff members:  10 with training using the system, 7 without training |
| 2017 | United Kingdom | Snowden A; Kolb H | Two years of unintended consequences: introducing an electronic health record system in a hospice in Scotland | EHR system | Survey (containing the System Usability Scale) at 4 time points during implementation  Multiprofessional focus group | Hospice staff (baseline, second, third and final survey):  Total staff: 55 48 36 55  Admin/Support staff: 2 2 1 5  Care assistant: 12 6 3 16  Chaplain: 1 1 1 0  Doctor: 2 2 3 4  Manager: 3 2 4 1  Nurse: 28 26 17 23  Occupational therapist: 1 1 2 2  Pharmacist: 1 2 1 0  Physiotherapist: 1 0 3 1  Social worker: 1 2 1 0  Other: 2 3 0 3 |
| 2012 | USA | Sockolow PS; Bowles KH; Lehmann HP; Abbott PA; Weiner JP | Community-based, interdisciplinary geriatric care team satisfaction with an electronic health record: A multimethod study | EHR | Satisfaction survey  Observation, shadowing  Interviews | 39 clinicians (survey) (characteristics for 37 clinicians):  Age: <25 years 5 (13%); 25–34 years 5 (13%); 35–44 years 5 (13%); 45–54 years 13 (35%); 55–64 years 9 (24%)  Sex: Male 4 (11%); Female 33 (89%)  Occupation: Nurse 10 (27%); Nurse practitioner 9 (24%); Licensed practical nurse 5 (13%); Physical therapist 4 (11%); Occupational therapist 3 (8%); Physician 3 (8%); Social worker 2 (5%); Nurse administrator 1 (3%)  Healthcare years: 1–10 years 6 (16%); 11–20 years 9 (24%); 21–30 years 16 (43%); >30 years 6 (16%)  Computerized physician order  entry/EHR use: 0 years 16 (43%); 1–3 years 5 (13%); 4–6 years 6 (16%); 7–9 years 5 (13%); >9 years 2 (5%): Missing 3 (8%)  Computer knowledge: Below average 4 (11%); Average 20 (54%); Above average 9 (24%); Advanced 4 (11%)  15 clinicians (observation)  10 clinicians (observation after 6 months)  5 clinicians (interviews in time 1)  6 clinicians (interviews in time 2) |
| 2012 | Sweden | Stevenson JE; Nilsson G | Nurses’ perceptions of an electronic patient record from a patient safety perspective: A qualitative study | EPR | Focus group interviews | 21 registered nurses:  5 from medical units; 6 from surgical units; 4 from an orthopedic unit; 6 form a stroke unit  All women  Age: 20–29 years (n = 4), 30–44 years (n = 11), >45 years (n = 6)  Years in nursing: <2 years (n = 1), 2–5 years (n = 5), 5–15 years  (n = 10), >15 years (n = 5)  Use of the current integrated EPR for 12–18 months |
| 2018 | Canada | Strudwick G; McGillis Hall L; Nagle L; Trbovich P | Acute care nurses’ perceptions of electronic health record use: A mixed method study | EHR | Cross-sectional survey (phase 1)  Focus groups (phase 2) | 133 registered nurses (phase 1):  Sex: Female 121 (90.9%); Male 12 (9.1%)  Age: m=35.2 years (SD=9.7)  Working as a nurse: m=10.9 years (SD=8.8)  Country of education: Canada 109 (82%); Other 9 (6.8%); Unknown 15 (11.2%)  Informatics training: Yes 20 (15%); No 113 (85%)  Unit: Medical 66 (49.6%); Surgical 67 (50.4%)  Experience using an EHR: m=10.9 years (SD=3.8)  Experience using another EHR: Yes 47 (35.3%); No 83 (62.4%); Unknown 3 (2.3%)  Employment status: Full time 106 (79.7%); Part time 24 (18%); Casual 2 (1.5%); Unknown 1 (0.8%)  6 nurses (phase 2) |
| 2020 | Canada | Tazzeo C; Pritchard JM; Papaioannou A; Adachi JD | Promoting Osteoporosis Best Practices: A New Electronic Medical Record Tool | Tool integrated into an EMR (Osteoporosis and Falls Assessment Form) | Demonstration session  Chart-audit  Paper-based survey | 37 family physicians:  23 completed the survey |
| 2021 | USA | Thayer JG, Ferro DF, Miller JM, Karavite D, Grundmeier RW, Utidjian L, Zorc JJ | Human-centered development of an electronic health record-embedded, interactive information visualization in the emergency department using fast healthcare interoperability resources | EHR in emergency department | Cognitive task analysis, design, postimplementation feedback | 2 attendings, 4 fellows, 3 advanced practice nurses, 2 nurses |
| 2016 | Canada | Tran K; Leblanc K; Valentinis A; Kavanagh D; Zahr N; Ivers NM | Evaluating the Usability and Perceived Impact of an Electronic Medical Record Toolkit for Atrial Fibrillation Management in Primary Care: A Mixed-Methods Study Incorporating Human Factors Design | EMR toolkit for atrial fibrillation | Usability testing:  Observations, think-aloud, using the toolkit with test patients (phase 1)  Observations, interviews, using the toolkit with actual patients  Usability surveys | 14 primary care clinicians (usability testing):  13 family physicians, 1 nurse practitioner  5 family physicians, 1 nurse practitioner (phase 1)  8 family physicians (phase 2)  12 participants (survey):  8 family physicians, 2 nurses, 1 nurse practitioner, 1 pharmacist |
| 2019 | USA | Wang X; Kim TC; Hegde S; Hoffman DJ; Benda NC; Franklin ES; Lavergne D; Perry SJ; Fairbanks RJ; Hettinger AZ; Roth EM; Bisantz AM | Design and Evaluation of an Integrated, Patient-Focused Electronic Health Record Display for Emergency Medicine | EHR display for emergency medicine | Concept design meeting with experts  Task performance  System Usability Scale; presentation of scenarios to perform using prototype display, task completion and usability questionnaire SUS | 20 clinicians:  10 nurses, 2 with charge nurse position  Age: m=33.2 years (24-54)  Gender: 9 female, 1 male  Years in position: median 4.25 (1-15)  10 providers (5 attending physicians, 4 residents, 1 physician assistant):  Age: m=33,7 years (28-41)  Gender: 8 female, 2 male  Years in position: median 3 (1-14)  19 reported to be expert or proficient in least two systems  all of them with experience working with an EHR |
| 2019 | France | Wawrzyniak C; Marcilly R; Baclet N; Hansske A; Pelayo S | EHR usage problems: A preliminary study | EHR | Interviews | 9 participants:  1 laboratory pharmacist, 1 clinical pharmacist, 1 anesthetist, 1 radiologist, 1 infectious disease specialist, 1 cardiologist, 1 emergency physician, 2 neurologists |
| 2015 | USA | Zhu X; Cimino JJ | Clinicians’ evaluation of computer-assisted medication summarization of electronic medical records | Computer-assisted medication summarization of EMRs | Interviews  Observations (clinical tasks) | 6 medical residents |
