# Supplementary material for: Involving Health Care Professionals in the Development of Electronic Health Records: Scoping Review
Source: JMIR Hum Factors. 2023 Jul 10;10:e45598. doi: 10.2196/45598 (PMC10366971; doi:10.2196/45598)
Supplement: Multimedia Appendix 2 [file humanfactors_v10i1e45598_app2.docx]

Involving Healthcare Professionals in the Development and Evaluation of Electronic Health Records – A Scoping Review

# Appendix 1: Search string

| **Plattform** | **String** | **Filters** | **Expanders** |
| --- | --- | --- | --- |
| **PubMed** | ("electronic health record*"[All Fields] OR "computerized medical record"[All Fields] OR "computerised medical record"[All Fields] OR "electronic patient record*"[All Fields] OR "computerised patient record*"[All Fields] OR "computerized patient record*"[All Fields] OR "electronic medical record*"[All Fields] OR "Electronic Health Records"[MeSH Terms]) AND ("participatory design"[All Fields] OR "participatory research"[All Fields] OR "cocreat*"[All Fields] OR "co-creat*"[All Fields] OR "codesign"[All Fields] OR "co-design"[All Fields] OR "collaborative design"[All Fields] OR "cooperative design"[All Fields] OR "coproduc*"[All Fields] OR "co-produc*"[All Fields] OR "consumer participation"[All Fields] OR "participatory development"[All Fields] OR "development"[All Fields] OR "usability"[All Fields] OR "testing"[All Fields] OR "user engag*"[All Fields] OR "user experience"[All Fields] OR "user involv*"[All Fields] OR "user participat*"[All Fields] OR "user-centered design"[All Fields] OR "user-centred design"[All Fields] OR "ux design"[All Fields] OR "usability study"[All Fields] OR "user centered design"[MeSH Terms]) | Abstract Available, English, from 2011 - 2021 | - |
| **CINAHL** | ("electronic health record*" OR "electronic patient record*" OR "computerised patient record*" OR "computerized patient record*" OR "electronic medical record*" OR (MH "Electronic Health Records+")) AND ("participatory design" OR "participatory research" OR "cocreat*" OR "co-creat*" OR "codesign" OR "co-design" OR "collaborative design" OR "cooperative design" OR "coproduc*" OR "co-produc*" OR "consumer participation" OR "participatory development" OR "usability" OR "testing" OR "user engag*" OR "user experience" OR "user involve*" OR "user participat*" OR "user-centered design" OR "user-centred design" OR "ux design" OR "usability study" OR (MH "Consumer Participation") OR (MH "Usability Study")) | Abstract Available; Published Date: 20110101-20211231 | Apply equivalent subjects; Narrow by Language: english; Search modes: Boolean/Phrase |
| **Google Scholar** | "electronic health record" OR "electronic medical record" AND "participatory design" OR "participatory research" OR "co-design" OR "collaborative design" OR "participatory development" OR "usability testing" OR "user experience" OR "user-centered design" | 2016 - 2021 |  |
| **Scopus** | ( ALL ( {electronic health record} OR {electronic patient record} OR {computerised patient record} OR {computerized patient record} OR {computerised medical record} OR {computerized medical record} OR {electronic medical record} ) AND ALL ( {participatory design} OR {participatory research} OR {cocreation} OR {co-creation} OR {codesign} OR {co-design} OR {collaborative design} OR {cooperative design} OR {coproduction} OR {co-production} OR {consumer participation} OR {participatory development} OR {usability} OR {testing} OR {user engagement} OR {user experience} OR {user involvement} OR {user participation} OR {user-centered design} OR {user-centred design} OR {ux design} OR {usability study} ) ) | document type: "ar" OR "re", publication date: 2011-2021, English |  |

# 
